# Computational Nutrition in Practice: Challenges and Opportunities From an Early-Career Perspective^☆^

**DOI:** 10.1016/j.tjnut.2026.101387

**Published:** 2026-02-03

**Authors:** Mattea Müller, Madeline Bartsch, Jan Voges

**Affiliations:** 1 Department of Clinical Data Science, Peter L. Reichertz Institute for Medical Informatics of TU Braunschweig and Hannover Medical School, Hannover Medical School, Hannover, Germany; 2 Department of Computational Biology of Infection Research, Helmholtz Centre for Infection Research, Braunschweig, Germany; 3 Department of Nutritional Physiology and Human Nutrition, Institute of Food Science and Human Nutrition, Leibniz University Hannover, Hannover, Germany; 4 NutritionLab, Faculty of Agricultural Sciences and Landscape Architecture, Osnabrück University of Applied Sciences, Osnabrück, Germany; 5 Institute of Information Processing, Leibniz University Hannover, Hannover, Germany; 6 L3S Research Center, Leibniz University Hannover, Hannover, Germany; 7 Lower Saxony Center for Artificial Intelligence and Causal Methods in Medicine (CAIMed), Hannover, Germany

**Keywords:** computational nutrition science, artificial intelligence, multiomics integration, early-career researchers, open science

## Abstract

Computational approaches are transforming nutrition science by integrating data from wearables, digital health platforms, and multiomics technologies to unravel complex diet–health interactions. Traditional statistical models cannot adequately capture the temporal, nonlinear, and individual variability inherent in such data. Computational nutrition, integrating data science, machine learning, and systems modeling, has therefore emerged as a distinct and rapidly developing field. Landmark studies have demonstrated its potential to improve dietary assessment, predict metabolic responses, and design personalized interventions. From an early-career perspective, however, the rise of computational nutrition also exposes structural and educational gaps. Early-career researchers often encounter fragmented training, limited mentorship, and restricted access to interoperable data and computational infrastructure. Empowering early-career researchers through integrated curricula, equitable data access, and recognition of interdisciplinary contributions will be essential for ensuring that computational nutrition evolves into a transparent, reproducible, and inclusive discipline capable of advancing both personalized and population-level nutrition.

## Introduction

Nutrition science is inherently interdisciplinary, drawing from biology, medicine, informatics, and the social sciences to understand how diet interacts with metabolism and health. The field is currently extended by new methodological innovations such as high-frequency sensing (e.g., continuous glucose monitoring and activity tracking), digital logging tools, and high-throughput multiomics profiling [1]. These developments are reshaping how dietary exposures and physiological responses are measured and interpreted, but they also expose the limits of conventional analyses for capturing nonlinearity, time dynamics, and interperson variability [2]. Computational nutrition has emerged as a new and expanding field that aims to integrate diverse data and modeling frameworks, ranging from statistical and causal inference to simulation and machine learning (ML), to describe, predict, and ultimately simulate the effects of diet on human physiology and health [3]. However, this emerging field faces challenges in the form of heterogeneous and nonstandardized data, data size–dimensionality mismatch, underreported preprocessing and validation, limited code and pipeline sharing, as well as concerns regarding interpretability and clinical credibility. Early-career researchers (ECRs; i.e., PhD students and early postdocs) are particularly affected by these challenges, often working in this interdisciplinary field without the essential training, mentorship, or infrastructure. In this *Perspective*, we outline core computational approaches and landmark interventional studies that demonstrate translational potential and articulate the structural barriers facing ECRs, alongside concrete potential opportunities. Our goal is to raise awareness of the importance of interdisciplinary fluency and the ability to collaborate and critically evaluate emerging computational methods, so that computational nutrition matures as a rigorous, transparent, and inclusive discipline.

## Computational methods and landmark studies in computational nutrition science

Computational nutrition science brings together methods from statistics, epidemiology, computer science, and systems biology to capture better and explain the complexity of human dietary responses [4]. Over the past decade, progress has been driven by 3 intertwined developments: the availability of high-resolution and multimodal data, the use of models capable of capturing nonlinear and temporal dynamics, and the shift toward personalized prediction and simulation. A series of landmark studies illustrates how these elements come together in practice.

Early computational work in nutrition relied on mechanistic and statistical models, such as differential equations and mixed-effects frameworks, to simulate nutrient metabolism and postprandial dynamics [5]. These models highlighted the value of representing physiology as a dynamic system, but were limited by the amount and type of data available. With technological advances and increasing digitalization, diet, multiomics, clinical, and sensor-derived data have become far more accessible, making it feasible to combine diverse features at scale. The landmark study by Zeevi et al. [6] demonstrated this shift by integrating dietary information, microbiome composition, lifestyle factors, and clinical biomarkers to predict individual postprandial glycemic responses. Follow-up randomized trials confirmed that diets guided by such models could improve glycemic control in prediabetes and newly diagnosed type 2 diabetes [7,8]. Since then, computational personalization has expanded further. Adaptive digital twin approaches continuously update model predictions using ongoing sensor and behavioral data, enabling dietary guidance that responds dynamically to a person’s physiology over time. A recent trial demonstrated significant improvements in cardiometabolic markers using this type of real-time personalization [9]. In parallel, advances in deep learning have broadened what can be measured efficiently. Image-based dietary assessment systems can now automatically identify foods and estimate portion sizes [10], and foundation models trained on continuous glucose monitoring data capture subtle temporal patterns that generalize across populations [11]. Multiomics deep-learning approaches similarly integrate molecular and routine clinical features to support early prediction of chronic disease risk [12], illustrating how computation increasingly enables both richer measurement and more precise forecasting.

Computation has also opened the door to designing rather than merely predicting dietary interventions. A notable example is the randomized trial by Tunali et al. [13], which applied a Monte-Carlo–based optimization framework to generate personalized, microbiome-guided diets for individuals with irritable bowel syndrome. These personalized diets achieved clinical improvements comparable to the highly restrictive low-fermentable oligosaccharides, disaccharides, monosaccharides, and polyols diet while being less burdensome for patients. Looking ahead, generative approaches such as variational autoencoders and diffusion models may allow researchers to simulate counterfactual dietary scenarios *in silico*, analogous to how generative models are already used to model biological or clinical perturbations in adjacent fields [14].

Alongside predictive and design-oriented approaches, computational tools are increasingly being used to understand *why* individuals differ in their responses to diet. Unsupervised learning has revealed latent metabolic subgroups, or “metabotypes,” that respond differently to identical dietary exposures [15], helping explain heterogeneity observed in intervention studies. Causal inference and individualized treatment-effect estimation could extend this further by identifying which dietary strategies are most effective for whom [16]. In the Preventing Overweight Using Novel Dietary Strategies (POUNDS lost) trial, Hamaya et al. [17] applied causal forests to uncover personalized effects of high- compared with low-fat diets that were obscured in population-level analyses. Hybrid causal–LLM approaches take this idea in a new direction by using automatically inferred causal graphs to generate personalized food recommendations [18].

Complementing these developments are advances in temporal modeling. Dynamic Bayesian networks, recurrent neural networks, and transformer-based architectures [18,19] are increasingly used to capture how diet–health relationships evolve longitudinally and to estimate risk trajectories rather than single endpoints. These models handle the irregular, high-frequency, and context-dependent nature of many nutrition datasets, offering a more realistic representation of real-world physiology.

Collectively, these methodological developments mark a shift from static, population-level analysis toward dynamic, multimodal, and personalized modeling. Implementing such approaches, however, requires far more than algorithmic expertise. Robust workflows spanning programming, data stewardship, and reproducible research practices are essential. As datasets become increasingly heterogeneous and analyses more data-intensive, researchers must also manage metadata alignment, version-control preprocessing steps, and ensure interpretability and transparency. For ECRs in particular, sustaining these practices is challenging given the limited access to standardized training, interoperable datasets, and dedicated computational infrastructure.

## The ECR perspective on Limitations and Challenges in Computational Nutrition Science

**FIGURE 1:**
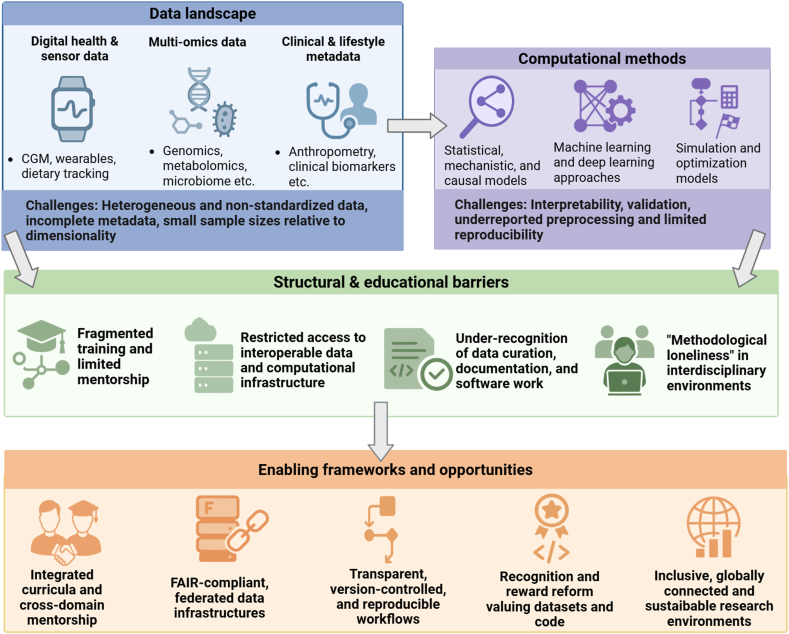
Overview of the challenges and opportunities of computational nutrition research from an early-career perspective. Digital health data, multiomics profiles, and clinical and lifestyle information form a heterogeneous and often nonstandardized data landscape feeding into diverse computational approaches. Early-career researchers must navigate fragmented training pathways, limited access to interoperable data and computational infrastructure, and the under-recognition of data curation and software work, often leading to methodological loneliness in interdisciplinary settings. Integrated cross-domain curricula, Findable, Accessible, Interoperable, Reusable (FAIR)–aligned data infrastructures, reproducible workflows, and reformed recognition and reward systems offer pathways to strengthen capacity and support sustainable career development in computational nutrition (created in BioRender. https://BioRender.com/ryc3q8d).

The expanding methodological landscape, therefore, exposes a widening gap between what is analytically possible and what is feasible for researchers to implement in practice, highlighting the need for institutional support, interdisciplinary teams, and shared computational resources rather than relying on individual upskilling alone (Figure 1).

### Usability and interoperability gaps

For many ECRs, the first obstacle is not analysis but simply acquiring usable data. Most do not generate large datasets themselves and therefore depend on external sources. Because many nutrition-related data types, including high-frequency behavioral records and multiomics profiles, are sensitive and in some cases re-identifiable, “open” data sharing in practice often relies on controlled access models such as secure data environments, governed data-use agreements, or tiered access systems which aim to balance Findable, Accessible, Interoperable, Reusable (FAIR) principles with privacy and regulatory requirements. In practice, access to these high-quality nutrition datasets requires financial resources, formal collaborations, or institutional affiliations that ECRs may not have. Even infrastructures intended to improve accessibility, such as nonprofit biobanks, including the Dutch LifeLines cohort [20], NIH Nutrition Science Data and Biospecimen Portal [21], or the Pan-European Food Nutrition Security-Cloud [22], still involve substantial administrative steps, limited datasets, or constraints on reuse. When datasets are obtained, their practical utility is frequently undermined by inconsistent food coding systems, shifting nutrient definitions, missing or incomplete metadata, and proprietary or poorly documented file formats. These issues make it difficult for ECRs to combine cohorts, reproduce published pipelines, or build on existing work, illustrating how data access and data usability remain deeply intertwined challenges in computational nutrition [23].

### Computational capacity and infrastructure inequities

Even when data are available, infrastructure limitations frequently restrict the capacity to analyze them. Many ECRs operate in departments without high-performance computing, secure data servers, or licensed analytical software. Cloud or database access fees, institutional restrictions, and limited IT support often confine researchers to small, local datasets, undermining scalability and reproducibility. Compared with fields such as systems biology or genomics, nutrition research remains under-resourced in computational infrastructure and lacks sustained funding for data management and software engineering positions [24]. For early-career investigators, these constraints translate directly into restricted analytical independence and limited opportunities to collaborate internationally.

### Skill and training deficits in an interdisciplinary field

The expansion of computational nutrition has outpaced the training structures required to support it. Most nutrition curricula still provide minimal exposure to data science, programming, or ML. As a result, many doctoral students and postdocs rely on self-directed online courses or informal mentorship to acquire computational skills, which may impede research quality [25]. It is important to note that ECRs may have highly heterogeneous backgrounds. Some come from nutrition, epidemiology, or clinical sciences with limited quantitative training, whereas others may already have strong foundations in statistics, computer science, or engineering. This variability does not diminish the structural challenges described below; rather, it underscores them. Even for technically strong ECRs, gaps in domain-specific training, access to interdisciplinary mentorship, and a lack of unified curricular frameworks make it difficult to navigate the field effectively. Further, the expertise needed for modern, data-intensive research extends well beyond scripting: it includes database management, metadata alignment, reproducible workflow design, and ethical data governance [26]. Even experienced computational epidemiologists struggle with model interpretability and bias [27]. Expecting nutritionists trained primarily in physiology or public health to independently master these domains risks shifting the burden of infrastructure development onto individuals, most of them in their early-career stages. A more sustainable solution is to foster hybrid research ecosystems embedding computational scientists alongside nutrition experts through co-mentorship, cross-faculty doctoral programs, and dedicated data teams [28,29]. These collaborative models are well established in genomics and systems biology but remain underdeveloped in nutrition science.

### Toward hybrid and FAIR research ecosystems

Adherence to FAIR (Findable, Accessible, Interoperable, Reusable) principles [30] is improving through initiatives such as Food Nutrition Security-Cloud, yet FAIR compliance rarely extends to analytical models. Preprocessing scripts, feature definitions, and trained model parameters are seldom archived or version-controlled, meaning that even open-code studies cannot be replicated without the underlying data. FAIR must encompass computational workflows as well as datasets if reproducibility is to be achieved [24]. Implementing this standard requires institutional investment in repositories, metadata curation, and research-software engineers, resources often unavailable to ECRs. Hybrid ecosystems could help alleviate these challenges by distributing technical expertise across teams, allowing nutrition researchers to focus on study design and interpretation while computational experts manage pipelines and FAIR compliance. However, such models require rethinking how authorship, funding, and credit are distributed in collaborative science.

### Structural and cultural barriers in academia

Beyond technical limitations, the academic culture surrounding computational nutrition remains a major obstacle. Fixed-term, grant-dependent contracts prioritize rapid publication and novelty over the slower, foundational work of data harmonization and software maintenance. The labor that sustains computational research (e.g., data cleaning, documentation, and pipeline upkeep) remains largely invisible in authorship and promotion criteria [[31], [32]]. Interdisciplinary manuscripts often face protracted review cycles due to a shortage of reviewers fluent in both nutrition and computation, whereas mentorship for hybrid projects remains limited. These pressures contribute to what has been termed methodological loneliness, i.e., a sense of isolation among researchers working at disciplinary intersections without embedded data-science expertise or supportive peer communities [33]. Further, as noted in other areas of health research [34], the rapid expansion and public visibility of artificial intelligence have contributed to inflated expectations about what current models can reliably achieve. This environment can intensify feelings of overwhelm, particularly in the absence of structured training or mentorship. Addressing these structural barriers will require institutional recognition of interdisciplinary labor and new evaluation frameworks that reward openness, collaboration, and reproducibility.

## A Forward-Looking Vision

Despite these challenges, a number of promising initiatives are emerging to bridge disciplinary divides and strengthen capacity in computational nutrition, reflecting a growing emphasis on interdisciplinary training and data literacy within the profession [35,36]. Programs such as the NIH T32 Training in AI and Precision Nutrition Program [37] or the Marie Skłodowska-Curie NUTRIOME Doctoral Network [38] exemplify structured co-mentorship and cross-faculty collaboration that foster genuine dual literacy. Likewise, formal interdisciplinary curricula emerge within Europe and internationally and thus redefine nutrition education by embedding computational, ethical, and open-science training within core nutritional education. Together, these programs mark a cultural shift from individual upskilling to collective competence, where computational literacy and data stewardship are no longer niche skills but shared responsibilities within the research environment. For this transition to be sustained, institutions and funding agencies must move beyond isolated training opportunities toward the development of structured and equitable pathways for interdisciplinary education. This includes integrating computational modules into existing nutrition curricula, supporting hybrid doctoral programs co-supervised by domain and data-science mentors, and creating accessible micro-credential programs for practicing dietitians and researchers in under-resourced regions. Policy frameworks such as the European Commission European Research Area Policy Agenda on Reforming Research Assessment further consolidate the FAIRification of research by redefining what counts as scientific contribution [38]. Looking forward, computational nutrition will depend less on the invention of new algorithms than on the cultivation of a supportive and equitable research ecosystem. Building bridges between nutrition, data science, and computer science requires institutional commitment to interdisciplinary training, shared infrastructure, and reformed academic incentives. If realized, these changes could transform computational nutrition from a technically demanding niche into a model of collaborative, transparent, and inclusive science.

## Author contributions

MM, MB, and JV wrote the paper. MM had primary responsibility for final content. All authors read and approved the final manuscript.

## Data availability

Not applicable.

## Declaration of Generative AI and AI-assisted technologies in the writing process

During the preparation of this work the author(s) used ChatGPT5 and Google Gemini 2.5 Pro to correct grammar and language. After using this tool/service, the authors reviewed and edited the content as needed and take full responsibility for the content of the publication.

## Funding

MB was supported by the CARLA Talent Academy – Health and Living of Osnabrück University of Applied Sciences. MM was supported by the Joachim Herz Foundation (Add-on Fellowship for Interdisciplinary Life Sciences). This work is partially supported by the Ministry of Science and Culture of Lower Saxony through funds from the program Zukunft. Niedersachsen of the Volkswagen Foundation for the “CAIMed – Lower Saxony Center for Artificial Intelligence and Causal Methods in Medicine” project (grant number ZN4257).

## Conflict of interest

The authors have nothing to disclose.
